# Challenges and opportunities for improvement in the management and financing system of Health Promotion Offices in Hungary

**DOI:** 10.3389/fpubh.2023.1219186

**Published:** 2023-10-27

**Authors:** Krisztián Horváth, Gergo Túri, Csilla Kaposvári, Borbála Cseh, Csaba László Dózsa

**Affiliations:** ^1^Department of Public Health, Semmelweis University, Budapest, Hungary; ^2^Med-Econ Human Services Ltd., Budapest, Hungary; ^3^Synthesis Health Research Foundation, Budapest, Hungary; ^4^Faculty of Health Sciences, Doctoral School, University of Pécs, Pécs, Hungary; ^5^Doctoral School of Medical Sciences, Semmelweis University, Budapest, Hungary; ^6^Department of Theoretical Health Sciences, Faculty of Health Sciences, University of Miskolc, Miskolc, Hungary

**Keywords:** Hungary, public health, Health Promotion Offices, network, organization, model, management, finance

## Abstract

**Background:**

One hundred ten Health Promotion Offices (HPOs) have started operating in Hungary in response to public health challenges. Many of them have been active for almost 10 years, yet their operational experience has not been evaluated. The specific objectives of our study were: (1) to describe the current operational and funding system of HPOs, (2) to identify challenges related to the current management and funding practices, and (3) to formulate recommendations for improvement based on gathered experience and international experience.

**Design:**

In order to gain a deeper insight into the operational experience of HPOs, an online survey was conducted with the professional or economic managers of HPOs. A scoping review was carried out to gather international experiences about best practices to formulate recommendations for improvement in developing the operational and financing scheme for HPOs.

**Results:**

We found that current HPO network in Hungary faces three main challenges: a deficient management system, inflexible financing scheme, and unequal ability to purchase or provide services for the population.

**Conclusions:**

Based on the survey complemented by international experiences, we propose the overhaul of the professional management system and switching toa combination of fixed and performance-based financing scheme for the HPOs in Hungary.

## 1. Introduction

In the 2000s, the role and importance of health promotion activities in developed countries began to accelerate, mainly due to the pronounced shift in disease burden toward non-communicable, chronic diseases and rapidly aging populations, especially in Japan and the European Union (1, 2). A shift toward prevention focused, “proactive” healthcare system is unfolding, where mitigating risk factors are becoming more important, rather than “reactive” approach treating existing morbidities (3). Thus, initiatives, services and programmes that target lifestyle and health culture factors i.e., significant determinants of health, such as smoking, excessive alcohol consumption, physical inactivity, and unhealthy diet -, are gaining interest, and the interventions that can positively influence them at population level are becoming increasingly valued (4–7).

Hungary is a landlocked country in Central-Europe with a population of roughly 9.6 million (8). The country's healthcare system faces numerous challenges chiefly due to an aging population and lifestyle related, preventable non-communicable disease burden. Life expectancy at birth in Hungary lags the OECD average and neighboring Visegrad countries (9). Significant inequalities in health status and life expectancy by economic, social and geographical dimensions can also be identified inside the country (10, 11). In addition, the morbidity and mortality rates from cancer in Hungary are above the OECD and EU averages, and in some cases (e.g., lung cancer) showing one of the least favorable epidemiological characteristics in the world (12, 13). The healthcare workforce is aging at a faster pace than the general population, with around 10% vacancy in the GP practices already, while the migration of skilled labor further increases the strain on healthcare sector (14). In response to public health challenges, several government strategies and programmes were developed from the early 2000s alongside community-based initiatives such as community health plans and Health Promotion Offices (HPOs) (15–18).

Given that HPOs activities are focused on health promotion, it is important to highlight the Hungarian nomenclature. The Act CLIV of 1997 on health care regulates the health promotion activities performed within the territory of the country (17). The definition of health promotion and prevention in this Act together with its amendments underlies the activities of the HPO's. Section 37 (1) of this Act defines health promotion “as a process by which individuals increase their skills to improve their own health, gain the ability to maintain a healthy lifestyle and adapt to a changing environment. Health promotion compromises activities that aim at improving the population's knowledge of health, encouraging healthy behavior and preventing health-related harm and disease” (19). Furthermore, according to Section 37 (2) “Health promotion fields of action cover the development of individual capacities, the strengthening of community action, the creation and maintenance of a health-promoting environment, the formulation of health-promoting policies and the re-tuning of the health care system toward a prevention-oriented approach” (19). The definition of prevention covers the identification, assessment and communication, elimination of risk factors; early detection of disease susceptibility, health prevention in disease, and creating supportive community and environment.

The first two “pilot” HPOs were established in 2008 on the initiative of two mid-sized county centers and other public health actors (19). Based on the positive experience of the first two HPOs, further HPOs were established in two phases via EU grants: 60 HPOs were established in 2013–2015, mainly in the less economically developed regions of the country (20). Another 50 HPOs were established in 2018–2020, so in total 110 HPOs operated in Hungary at the end of 2022.

The key objective of establishing the HPOs was to provide a range of community-based disease prevention and health promotion services to the local population free of charge, co-financed by central budget and EU cohesion funds (17, 19, 20). A secondary objective was for the HPOs to provide an official institutional background for disease prevention and health promotion activities by actively operating and integrating health promotion organizations and professionals into a sustainable local network. In addition, maximizing the involvement of local stakeholders and opinion leaders in effectively implementing health promotion programmes in communities was also a goal.

Although 63% of Hungarian so-called small districts has HPOs in 2023, and a significant number of them have been in operation for almost 10 years, very few analyses of their operational experience have been carried out so far, and a few were exclusively published in Hungarian language (17–20). Since the HPOs generated many valuable experiences, challenges and opportunities that may be of interest to the international public health community, we conducted a comprehensive analysis of their network in our research. The results of our study can contribute to the development of the operational system of HPOs and can also serve as lessons learned for developing public health systems in other middle- and high-income countries, facing similar challenges across the globe.

The specific objectives of our mixed method research were as follows:

1. To describe the current operational and funding system of HPOs.
2. To identify challenges related to the operation and funding of HPOs.
3. To formulate recommendations to improve the operational model and financing of HPOs based on international experience.

## 2. Methods

Two different methodologies were used in this mixed-methods original research. An online questionnaire survey was conducted on the operational practices of HPOs, a literature review was conducted on the current management and financing practices of HPOs and another review on promising international practices.

### 2.1. The method of the online survey on the operational practices of HPOs

As previously only limited in-depth data and information were available on the operational practices of HPOs, firstly, an online survey was conducted in December 2021 among the Health Promotion Offices operating in Hungary in relation to 2021. A link to the online survey has been sent to the publicly available work email addresses of the HPOs' professional and economic managers. Only one manager from each HPO was asked to complete the questionnaire anonymously, but managers were able to discuss their responses with each other before completing it. The total number of respondents was 110. Thus, 100% of HPOs participated in the survey, making the sample representative of HPOs in terms of geographical distribution and HPO size. There was no duplicate completion—i.e., multiple completions from one HPO. The survey consisted of a questionnaire and an open-ended section. The open-ended questions gave opportunity for HPOs to reflect on experiences, perceived challenges in management, service portfolio, communication, intersectoral collaboration and the operational implications of the COVID-19 pandemic. The questionnaire section focused on the financing model and human resources (HR) aspects. HPOs were asked to summarize their 2021 budget by type of expenditure (such as salary costs, purchase of services, and rental costs). During our survey, only organization-level, otherwise public domain data was collected; as such, our research did not fall under any regulations relating to the Personal Data Protection Act or research ethical permission. Data provision during the survey was anonymous. Since the HPOs were initially established using EU-co funding, information about their operations is open for dissemination. The HR questionnaire section asked HPOs to provide anonymised data on the number of employees and their educational qualifications. Data were collected and managed using REDCap (Research Electronic Data Capture), a secure, web-based software platform to support data capture for research studies (21). The online survey was completed anonymously by the professional or economic manager of the HPOs, and all HPOs operating in 2021 participated in the survey. Data were extracted into an Excel spreadsheet, and the human resources and financing data were calculated using Excel syntaxes. The normal distribution of HR and financial variables (such as number of employees, revenues and expenditures by subtype) was tested using Kolmogorov-Smirnov and Shapiro-Wilk tests using SPSS Statistics Program v27. These showed that the HR and financial variables followed a normal distribution. Text responses from HPOs on operational practices and challenges were organized according to a coding frame with label definitions using Atlas.ti software. Two research team members did the coding in parallel, and the whole team interpreted the results.

### 2.2. The method of the review regarding the current management and financing system of the HPOs

**Figure 1:**
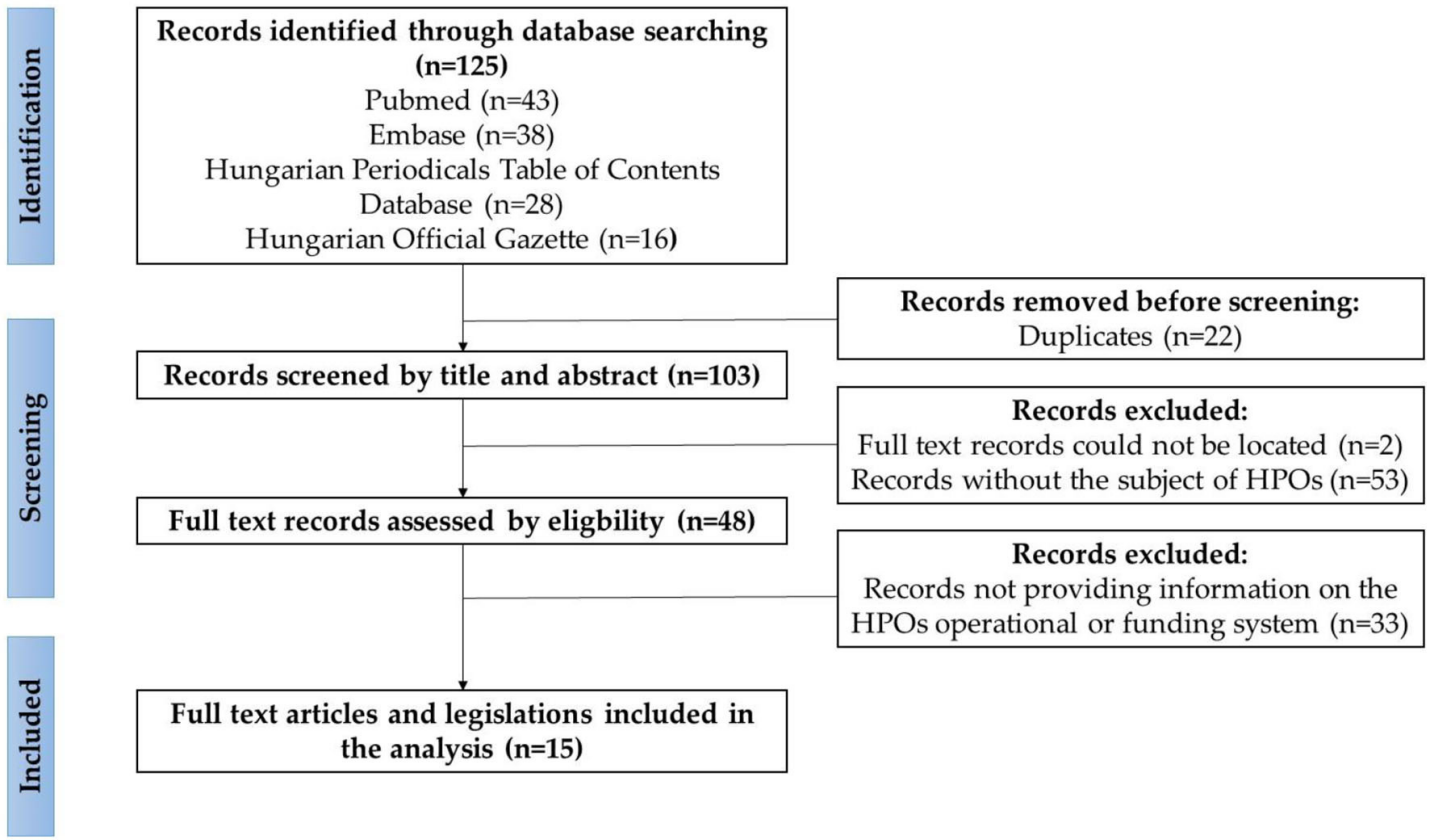
Flowchart of the results of the search on the current management and financing system of HPOs in Hungary, according to the PRISMA guideline (22). Sources: Authors, based on the PRISMA guideline (22).

For an overview of the current management and financing system of the Hungarian HPOs, a literature search was performed in January 2023 using the Embase database, the Hungarian Periodicals Table of Contents Database, the Hungarian Official Gazette, and the PubMed search engine, using the following keywords in Hungarian and English: health promotion, health promotion office, health promotion offices; health promotion network; Hungary (Figure 1).

The selection criteria were availability of full text publication, or legislation that should contain relevant information on the financing and management of HPOs in Hungary and that it should be fully accessible in Hungarian or English. Exclusion criteria were publications published before 2006, editorials, conference papers, commentaries, abstract only publications. The literature search resulted in 125 publications; after screening the titles, abstracts, and full text documents, 15 publications in Hungarian and legislations were included in the analysis. No relevant analysis published in English was identified in the search. Publications and legislations were organized according to a coding frame with label definitions using Atlas.ti software. Two research team members did the coding in parallel, and the entire team took part in the interpretation of the results.

### 2.3. The method of the scoping review regarding the promising international practices

A scoping review was carried out in January 2023 to gather insights about promising practices to formulate recommendations for improvement in developing the operational model and financing of HPOs. The scoping review was performed according to the PRISMA guideline of Tricco et al. (22). The study protocol specified the purpose of the study, the search strategy and keywords, the inclusion and exclusion criteria, the method of data selection and analysis, and the method of synthesis of the results.

**Figure 2:**
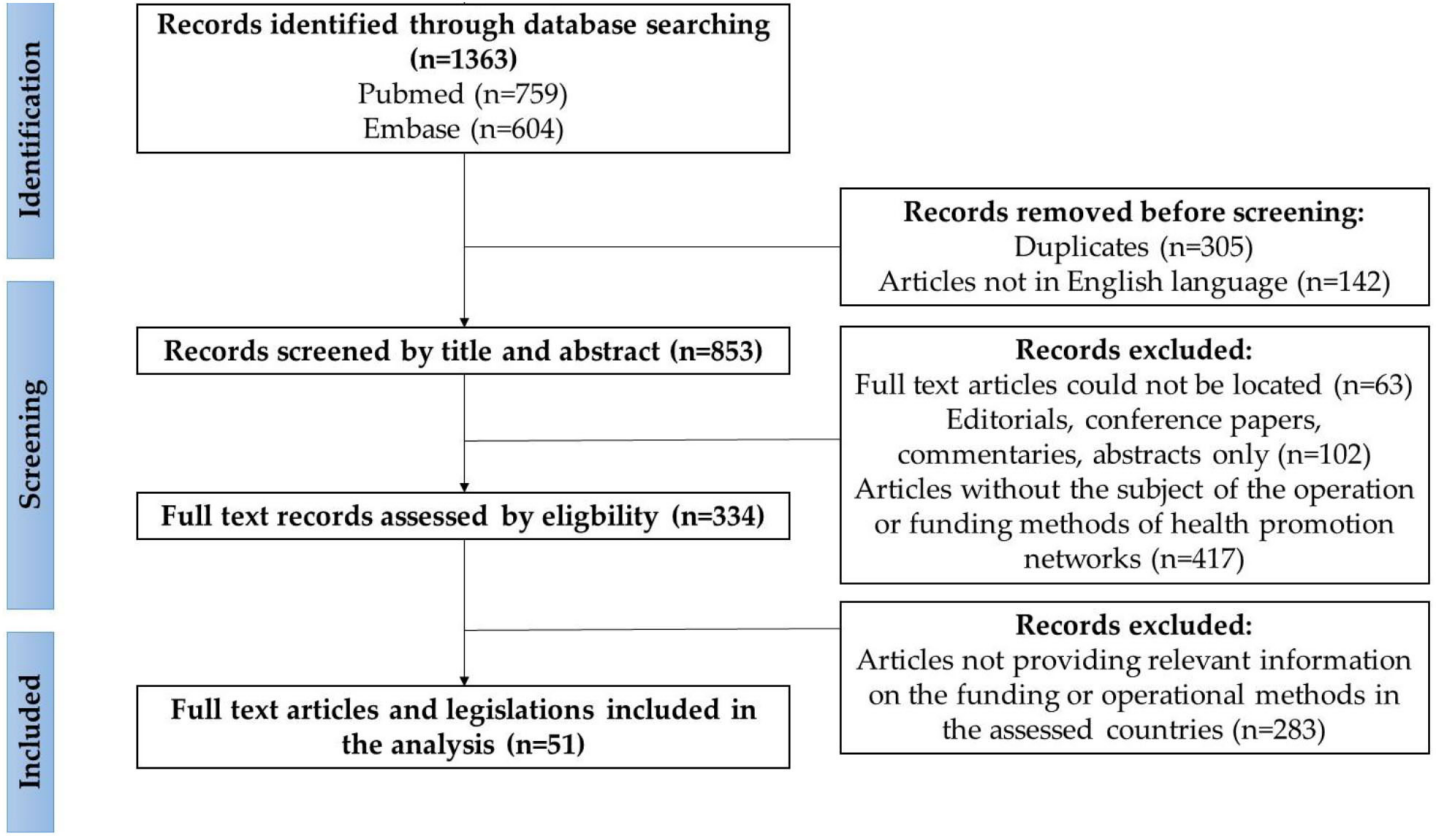
Flowchart of the results of the search on the promising international practices, according to the PRISMA guideline (22). Sources: Authors, based on the PRISMA guideline (22).

The literature search on public health systems operational models was performed with using the PubMed search engine querying the Embase database using the following keywords: public health, health promotion, system, network, model (Figure 2). The literature search on public health systems financing methods was performed with PubMed search engine on the Embase database using the following keywords: public health, health promotion, finance, financing, reimbursement. The literature search collected information on public health systems in 7 developed countries: the United Kingdom, Canada, Italy, Norway, Netherlands, New Zealand, and the USA. The selection criteria were that the full text publication should contain relevant information on the management and financing of public health systems in the countries concerned and that it should be fully accessible in English. Exclusion criteria were publications published before 2012, editorials, conference papers, commentaries, abstract only publications, or did not contain specific and relevant information on the public health systems of the countries under review. Finally, the literature search resulted in 1,363 publications; after screening the titles, abstracts and full text documents, 51 publications were included in the analysis. Publications on promising international practices were organized according to a coding frame with label definitions using Atlas.ti software. Three research team members did the coding in parallel, and the whole team interpreted the results and formulated recommendations for HPOs.

## 3. Results

### 3.1. The current operational and funding system of the Hungarian Health Promotion Offices

This section contains the results of the literature search in Hungarian and English on the current operational and financing system of HPOs. Fifteen publications in Hungarian and legal texts were used in the analysis.

**Figure 3:**
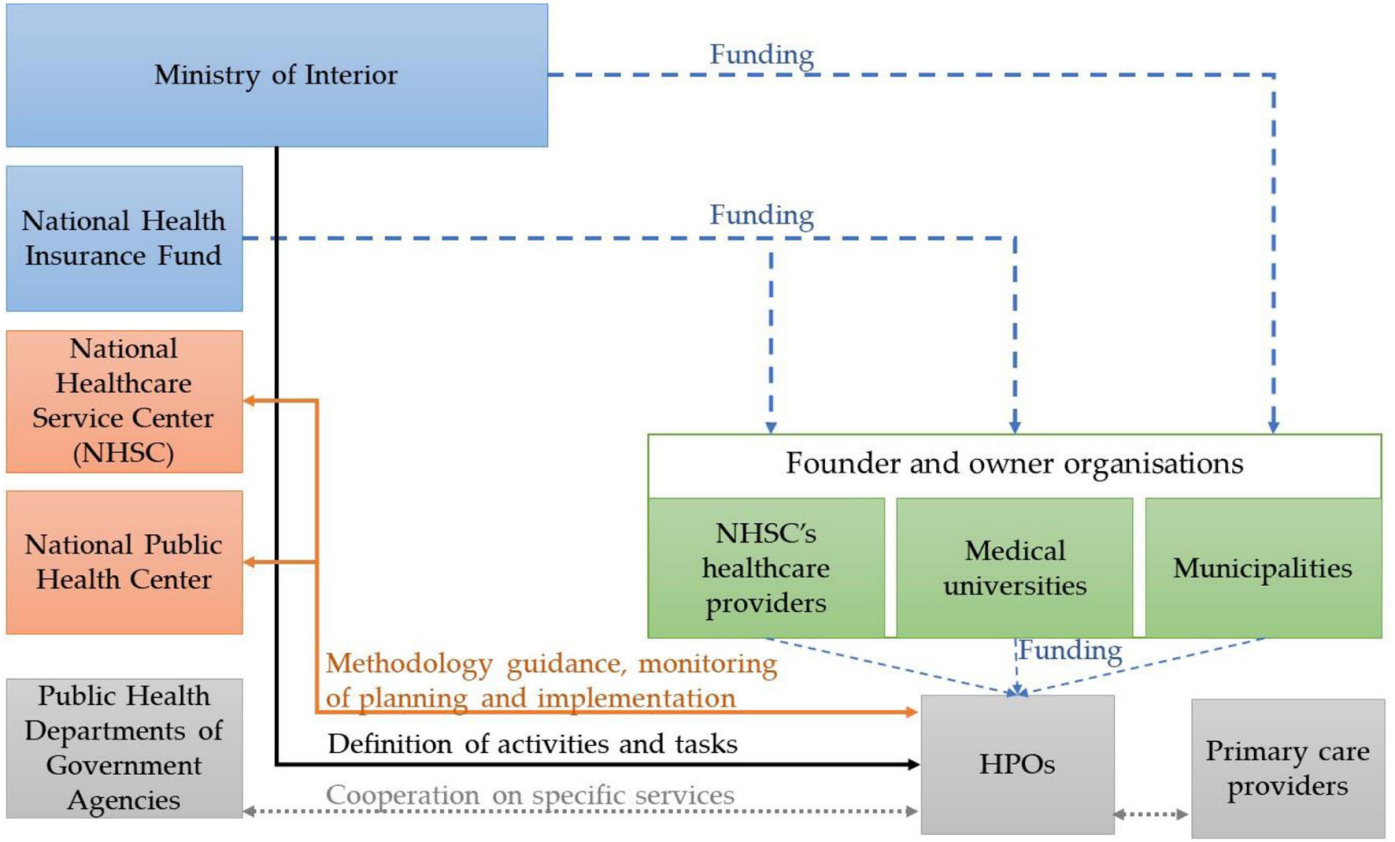
Simplified figure of the operational system of HPOs in Hungary. Sources: Authors.

In Hungary, the central government plays a crucial role in the workings of the public health system, as health-related legislation and policymaking at the national level are the responsibility of the Government and the Ministry of Interior (23). The Ministry of Interior determines the activities and tasks of the HPOs (Figure 3). The professional management, methodological support, planning of activities and monitoring of the implementation of the HPOs are shared between the National Public Health Center and the National Healthcare Service Center (24, 25). The Public Health Departments of Government Agencies, also at the district level, are responsible for organizing and coordinating public health screening, coordinating health care services, developing public health programmes in cooperation with the HPOs in their small districts (25). The HPOs also work in collaboration with primary care providers in their operational territory.

Since the establishment of HPOs in 2013 and 2018, the ministries and national institutes responsible for management have undergone major institutional reorganization almost every year (26–30). Limited methodological documents or publications are publicly available on HPOs' monitoring, performance assessment and financing methods. The key performance indicators (KPIs) defined are output indicators (such as the number of health promotion programmes and services delivered by the HPO, the number of populations involved in the programmes, and the number of partnerships established), and did not incentivize cost-effectiveness, or assessing the quality of services provided (17, 19, 31). The current funding uses a base funding method whereby each HPO has the same yearly budget.

The HPOs are responsible for the health promotion of the population in their small districts, and their activities can be grouped into four main categories: (a) individually oriented health promotion and prevention services; (b) community-based health services; (c) health communication, and (d) local partnership development (17–20, 31, 32). Under individually oriented health promotion and prevention services, HPOs carry out risk assessments of individuals (such as body mass index, waist circumference, smoking habits, alcohol consumption habits, and mental health). They also provide counseling and refer individuals to a general practitioner or specialist if necessary. For local community health promotion services, HPOs provide theme-, target group- and setting-specific community programmes and services. Examples of such services include school and workplace health promotion programmes, parenting skills training courses, sports clubs, depression and burnout prevention programmes, and health promotion initiatives for the older individuals and chronically ill (17, 20). HPOs are also responsible for implementing the specific elements of national strategies and public health programmes for a given year. These activities include programmes and services with a varying focus from year to year, in collaboration with different national and local partner organizations.

In the health communication tasks group, HPOs implement topic and target group-specific communication campaigns (online, radio and TV platforms) and participate in the local implementation of national public health communication campaigns. Regarding the local partnership development services, HPOs are responsible for initiating and managing district-level cooperation and intersectoral partnerships and supporting the development of municipal and district health plans (19). In addition, HPOs have an essential role in raising awareness of decision-makers on (mainly local) health-related issues. Their goal can be summarized as advocating “health in all policies” with every stakeholder in their network, continuously working on expanding said network.

To summarize, HPOs have a broad portfolio of services and partnerships; their governance system is multi-stakeholder with sometimes overlapping roles and their funding system is based on a simple base funding approach.

### 3.2. Results of the online survey on the operation and funding of HPOs in Hungary

This section presents data analysis from the online survey of 110 HPOs, which was designed to complement the knowledge of HPOs' operational and funding systems and to identify the issues and challenges perceived by HPOs.

#### 3.2.1. Human resources and financing mechanisms

According to the results of our online survey, the network of HPOs employed 832 people in 2021, with an average of 7.6 employees per HPO. HPO staff members' educational backgrounds were diverse, with the majority working in health, public health, social and other human services disciplines, with 55% of staff working full-time and 45% working part-time. The organizations that own HPOs can be grouped into three categories^1^: 41% of owners (46 HPOs) are National Healthcare Service Center (NHSC) healthcare providers, 2% of owners (2 HPOs) are medical universities and 57% of owners (62 HPOs) are local governments. The owner organizations are responsible for providing the operational requirements for the HPOs, such as infrastructure, devices and salary. The necessary funds for municipal HPOs are provided by the Ministry of Interior.

Regarding HPOs owned by medical universities and healthcare providers, operational funds are provided by the National Health Insurance Fund (NHIF). The HPOs are reimbursed by base financing method, meaning that each HPO receives a fixed annual amount of 62,500 EUR after their annual activity and budget plan is approved by the Ministry of Interior.^2^ However, while HPOs owned by healthcare providers and medical universities receive monthly funding, municipal HPOs receive the funding in a lump sum after a more extended administration period.

Based on the survey data, the total annual revenue of the 110 HPOs was 7.81 million EUR in 2021. The average annual revenue of HPOs was 71,000 EUR, and the median revenue was 62,500 EUR, which was in line with the statutory funding budget. This shows that 12% of the HPOs received “extra” financial support from their owning organizations, with an average of 4,000 EUR.

**Figure 4:**
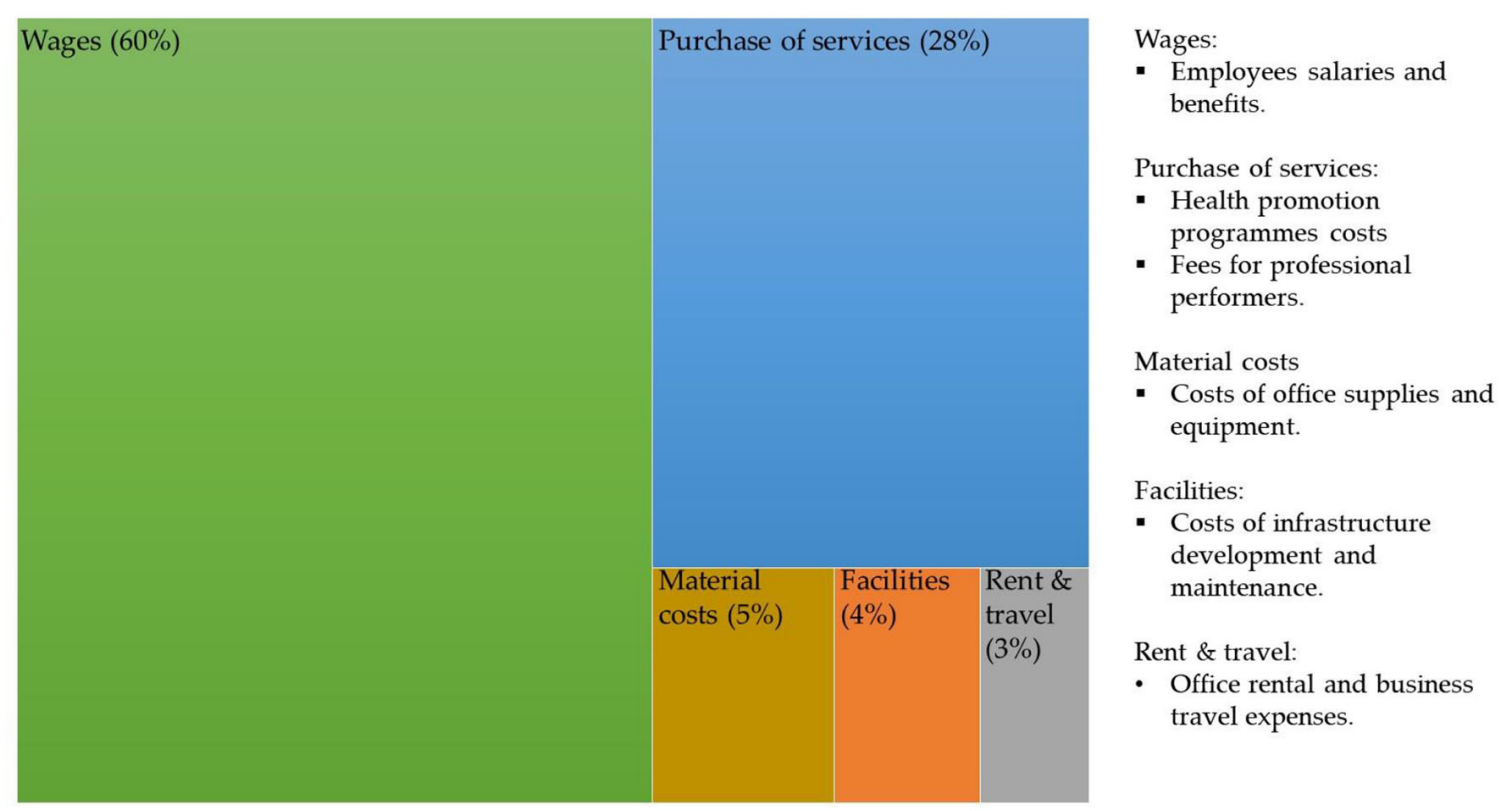
Distribution of the total annual expenditure of an average HPO in relation to the total annual revenue in 2021. Sources: Authors.

The total annual expenditure of HPOs in 2021 was HUF 7.81 million EUR, of which 60% (4.5 million EUR) was accounted for by wage costs, while 28% (2.17 million EUR) was for the purchase of services, 5% (387,500 EUR) for material costs, 4% (310,000 EUR) for infrastructure improvements and 3% (232,500 EUR) for rent and travel expenses (Figure 4).

However, the distribution of the different types of expenditure was heterogeneous among HPOs. While the average HPO spends 60% of its income on wage costs, some HPOs spent up to 85% or, in some cases, 18% of their income on wage costs. The same heterogeneity was found for HPOs' purchased services as a proportion of their total expenditure.

#### 3.2.2. Challenges related to the operation and funding of HPOs

**Figure 5:**
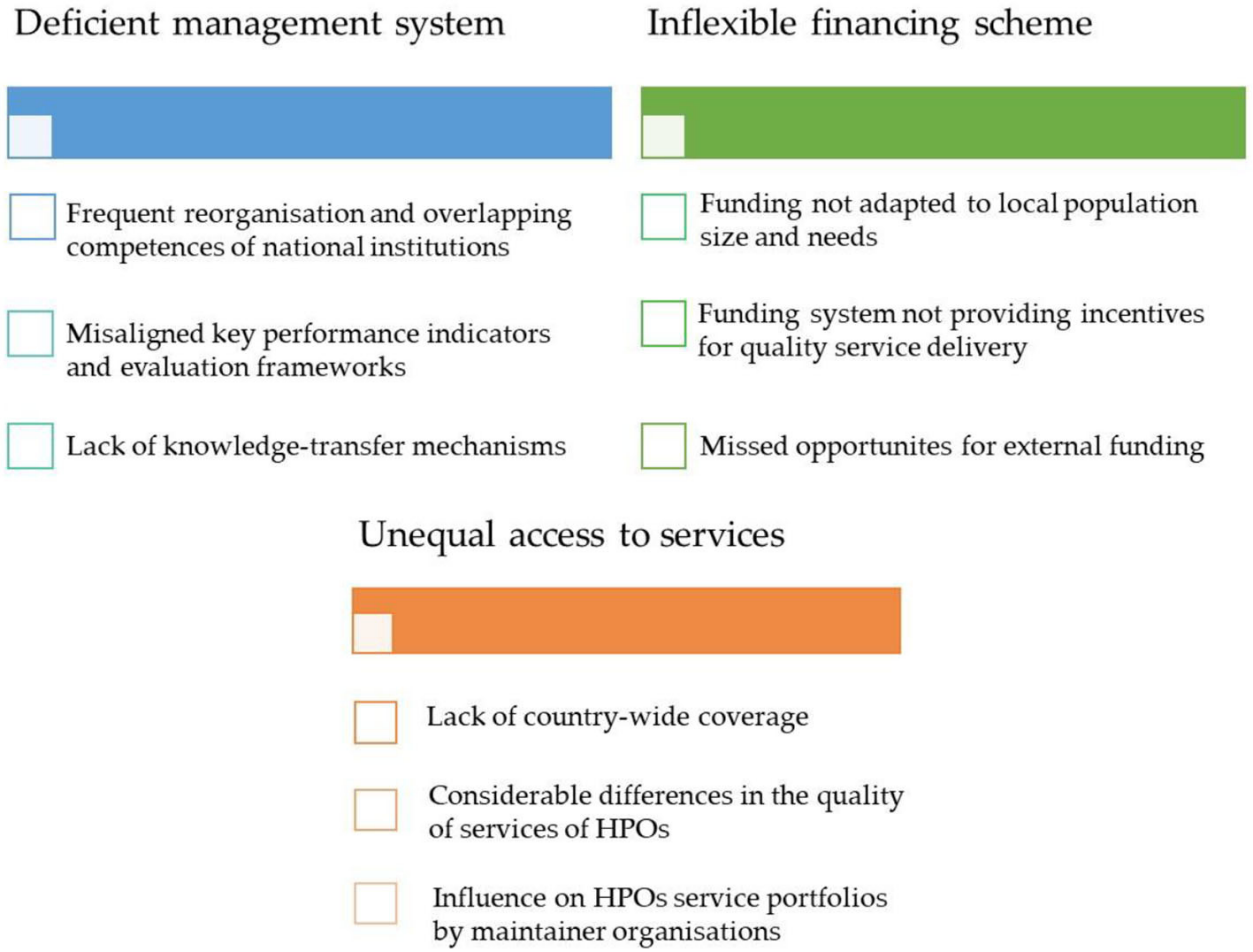
Main challenges related to the operation and funding of HPOs in Hungary. Sources: Authors.

Based on the results of the online survey, the current network of HPOs faces three main challenges: (a) a deficient management system, (b) inflexible financing scheme, and lastly but consequently (c) unequal capacity to purchase or provide services for the population in their respective jurisdiction (Figure 5). The issues related to these challenges are detailed below.

##### 3.2.2.1. Deficient management system

According to the online survey results, frequent reorganizations of the national institutions created overlaps in the tasks and competencies of these organizations. The day-to-day functioning of the HPOs was negatively affected by the changes in administrative rules, policy priorities and responsibilities resulting from the frequent restructurings. Apart from the issues at national level, attempt has been made to develop management and coordination at the county level via a 3-year pilot project, where a methodology has been developed and tested (32). According to several survey respondents, at the end of the pilot project, these initiatives were also discontinued, the generated insights and knowledge of the experts were not preserved, in addition the methodological improvements were not adapted by any national organization. Few respondents highlighted that, it was never defined whether HPOs should provide services with their own staff, or contract third-party providers, leading to a patchwork approach with significant heterogeneity.

HPO staff perceived the lack of knowledge-sharing mechanisms across the network as a problem, as well as the not up-to-date methodological guidance from the national level.

##### 3.2.2.2. Inflexible financing scheme

According to the results, the current base financing method does not take into account the size and socioeconomic or epidemiology characteristics of the population in the HPOs respective operational area. The funding mechanism does not differentiate between HPOs operating in various small districts: the service portfolio of an HPO operating in an economically developed urban environment with a younger age group should reflect the needs of the community, thus differ from an HPO operating in a less urbanized district with an aging and socioeconomically disadvantaged population. In addition, local health needs are less well identified, with only a small proportion (8%) of HPOs in our survey having developed or participated in the creation a community health plan. Moreover, significant differences can be identified in terms of the extra resources available based on the type of institutions responsible for the upkeep of HPOs. For example, a medical university or hospital with a larger institutional budget can support its HPO to a greater extent in terms of financial, methodological, health infrastructural, and human resources compared to HPO owned by a small town municipally with more limited resources.

According to several survey respondents, the operation of the funding system is not transparent, as the details of the HPOs' service procurement are not well regulated, it is challenging to control activities and costs. As a result, HPOs operate on a fixed monthly amount and, as payments do not take performance into account, the current practice encourages the spending of all the resources rather than focusing on increased efficiency or improved quality of services.

Other problems with the current funding method include the low level of external funding. According to the online survey, only 12% of HPOs could obtain extra financial support from their owner organization. Only three HPOs reported receiving other non-financial resources (such as in-kind contributions or voluntary work) from other organizations. This may be partly due to HPOs' lack of motivation to raise extra resources. However, the online survey results also suggest that the problem is due to HPOs' lack of knowledge of effective fundraising methods and lack of clarity on the rules for external fundraising.

##### 3.2.2.3. Unequal access to services

At present, HPOs operate in only 63% of the Hungarian small districts, representing only 55% of the Hungarian population. Thus, a significant part of the population still does not have access to the health promotion services that these organizations provide free of charge. According to the questionnaire results, HPOs have heterogeneous human resources with different skills and a heterogeneous service portfolio. Respondents found it particularly difficult to recruit suitably qualified staff for specific occupations (Supplementary Figure 1). Respondents also varied in assessing the difficulty of organizing health promotion services (Supplementary Figure 2). Most HPO managers identified incomplete and outdated protocols and guidelines as a problem for the inconsistent quality of health promotion programmes.

Furthermore, owner organizations can influence the service portfolio of HPOs, for example, by making the activities of HPOs belonging to health service providers focus on healthcare-related services rather than health promotion programmes. According to the survey results, the COVID-19 pandemic has also negatively impacted access to services and cooperation with local partners. Many health promotion programmes were canceled due to the restrictions, the vast majority in community settings (schools, workplaces). Although HPOs have been able to provide many services and programmes online, disadvantaged social groups with limited internet connection, such as the older individuals and the Roma communities, have had less access to these services. Having said that, nearly 15% of HPOs do not use online communication tools in their communication activities, which might make reaching younger generations difficult (Supplementary Figure 3). In addition, cooperation with GP practices was made difficult by the overburdened workload of GPs.

In summary, HPOs have a very diverse pool of workforce with a wide range of qualifications. Nearly 90% of their expenditure spent on salaries and the provision of services. The following issues have been identified in the HPOs' system: frequent reorganizations and overlapping competencies of national institutions; misaligned key performance indicators and evaluation frameworks; lack of knowledge-transfer mechanisms; funding not adapted to local population size and needs; funding system not providing incentives for quality service delivery; lack of country-wide coverage of services, differences in the quality of services.

### 3.3. Opportunities for improving the operational and financing scheme of Health Promotion Offices

Although HPOs face diverse challenges, they have become a cornerstone in the goal of improving local communities' health over the past decade. Targeted development of the management and funding system for HPOs might bring significant benefits to the Hungarian society. The countries included (Canada, Italy, Netherlands, New Zealand, Norway, United Kingdom, and USA) in the scoping review have advanced health systems, high GDP per capita and significant health expenditure as a share of GDP (Table 1). The promising international practices identified in the scoping review have been categorized according to the main challenges of HPOs. Recommendations were developed considering the international experience and domestic adaptability.

**Table 1:** Selected demographic, socioeconomic and health sector indicators of countries examined, 2022 or latest available year.

**Indicators**		**Canada**	**Italy**	**Netherlands**	**New Zealand**	**Norway**	**United Kingdom**	**USA**
Demographic factors	Population (million)	38	59	18	5.1	5.4	67	332
	Share of population over age 65 (%)	18.5	23.6	19.9	16	18.1	18.8	16.8
	Life expectancy at birth (years, total)	81.7	82.7	81.4	82.3	83.2	81	76.4
Socioeconomic factors	GDP per capita (USD)	58.348	51.790	69.578	51.455	114.932	53.942	76.360
	Poverty rate (%)	8.6	13.5	8.5	12.4	7.9	11.2	15.1
Health resources	Health spending as a share of GDP (%)	11.2	9	11.2	11.2	8	11.3	16.6
	Practicing doctors (per 1,000 inhabitants)	2.8	4.1	3.8	3.5	5,0.2	3.2	2.6

Sources: OECD (21).

**Figure 6:**
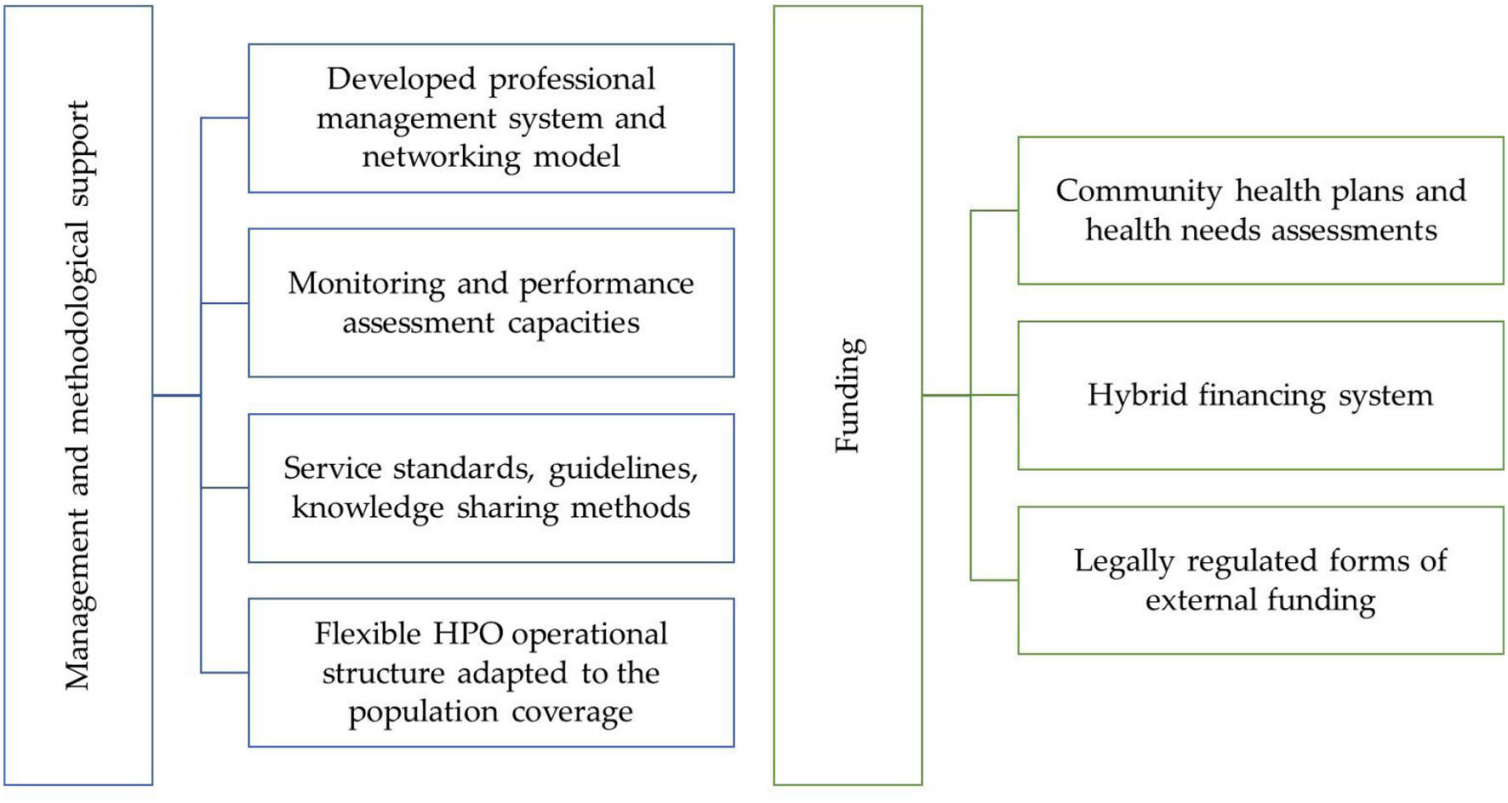
Opportunities for improvement in the management and financing scheme of Health Promotion Offices in Hungary. Sources: Authors.

We present our recommendations for improvement based on a review of public health systems in seven developed countries (Figure 6).

#### 3.3.1. Management and methodological support according to international experience

##### 3.3.1.1. Developed professional management system and networking model

The eight countries under review have developed a tiered management system based on the division of tasks and responsibilities within the administrative system (33–45). Organizations and actors have different functions but coordinate activities at each operational level. In all cases, the management of the public health system is at the national level. In several countries, a regional level can also be identified, where the organization is responsible for planning, organizing, and coordinating the activities of the health service providers in the region (34, 35, 37, 38, 43). Network-based cooperation is also common, whereby different organizations interested in promoting population health at the same or different operational levels form networks, partnerships, and collaborations. The development of networks and collaborations serves multiple purposes, such as joint development of knowledge and skills, sharing knowledge, information, and resources, and coordinating and harmonizing activities and services (36, 41, 43, 44, 46).

Recommendation for improvement 1: Based on these international experiences, it would be helpful to further develop the management system of HPOs in Hungary by eliminating the current duplication at the country level and adapting the network operating model and regional management functions.

##### 3.3.1.2. Monitoring and performance assessment capacities

Looking at the public health systems of the countries examined, several organizations operate at national and regional levels to monitor and analyse the factors and trends of the population health, health risks and unmet health burden (34, 35, 37–41, 43, 44, 47–49). These organizations also play an essential role in monitoring the activities and assessing the performance of service providers at the community level. Usually, public health priorities are set at national level, with implementation left to lower management levels (34, 35, 37–39, 41, 44). International experience shows that services can only be evaluated if adequate quality and quantity of data is available for analysis (48).

Recommendation for improvement 2: From the perspective of Hungarian HPOs, it is appropriate to develop the capacity and methodology of monitoring and evaluation organizations and to coordinate their activities at the system level. In addition, further developing the monitoring system would allow for broader monitoring, analysis, and evaluation of the impact of services and programmes provided by HPOs.

##### 3.3.1.3. Service standards, guidelines, knowledge sharing methods

Following the best international examples there are numerous examples of organizations at national or regional level responsible for formulating professional guidelines or standards for the provision of public health services (38, 41, 44, 47–54). These national organizations often provide professional support, monitor local providers' operations, and evaluate based on transparent indicators. The expected benefits of standardized service provision is the reduction of variation in service quality between geographical units and ensuring that evidence-based, effective services are provided to the population (48–50, 52, 53, 55, 56). It is important to note however that standardization is a sensitive topic in public health, and local needs have to be taken into account when developing or providing services. These organizations often play an essential role in facilitating two- way information and knowledge transfer (top-down or bottom-up approach), adapting evidence-based programmes developed by national-level organizations to the local level (36, 41, 49). In the countries we studied, universities and professional organizations, along with a variety of regional and community providers are involved in training public health professionals.

Recommendation for improvement 3: In light of these findings, in our view it is worth considering establishing an organization responsible for developing standards and guidelines for HPO services in Hungary, providing the human resources, tools and funding necessary for their operation, and ensuring that the guidelines are continuously updated. The standards developed by the national professional management body, can form the portfolio of services that HPOs can provide, building on international and national good practices. The involvement of HPOs in training health promotion practitioners would also be helpful, with the expected benefits of ensuring the supply of professionals.

##### 3.3.1.4. Flexible HPO operational structure adapted to the population coverage

In the United States of America, local health departments (LHDs) are responsible for providing public health services to the population (35, 57). LHDs are mainly responsible for serving a single county, usually with a population of tens to hundreds of thousands. However, some LHDs are responsible for areas with populations of up to one million. Both the services provided by LHDs and the number of staff they employ are commensurate with their area of operation: a county with a lower population is served by fewer workforce than average and a narrower, more specific portfolio of services. In comparison, a county with a higher population is served by more health professionals than average and has a broader portfolio of services.

Recommendation for improvement 4: Based on the example of the Local Health Departments in the US, it may be preferable for HPOs to operate in a relatively flexible structure, where the number of human resources and services provided by the organization is proportional relative to the population it aims to serve (35, 57).

#### 3.3.2. Funding

##### 3.3.2.1. Community health plans and health needs assessment

In the majority of the countries examined, a local or regional organization is responsible for developing community health plans and implementing, organizing, and coordinating the programmes described (35, 41, 43, 44, 58–61). The expected benefit of regularly updating these plans is that programmes implemented can respond to community needs and efficiently use community resources, with the active involvement of the population and the cooperation of community actors (62).

Recommendation for improvement 5: International experience suggests, it would be preferable for HPOs to play a more active role in preparing and implementing community health plans, with monitoring and methodological support provided by a national organization. The preparation of community health plans can also provide input data for planning the HPO's budget, as it is necessary to prepare a cost plan for the programmes and services included in the health plans both at national as at small district level (63, 64).

##### 3.3.2.2. Hybrid financing system

Due to their complex and intersectoral nature, the financing of health promotion programmes is far from straightforward (63, 64). Public health services are financed by diverse private and community actors globally (34, 35, 39, 41, 43, 44, 61, 65, 66). However, there are few practical guidelines and good practices available, albeit with different methodologies, in the international literature (63, 66–70). Over the past two decades, there has been increased interest in financing methods that support efficient financial activities and improved service quality (66). Therefore, payment systems have been developed that “pay for performance” by linking part of the payment for an activity to the results achieved or, when results are challenging to measure, by linking payment to the pursuit of processes that are assumed to lead to better results (e.g., service protocols or guidelines) (70).

Recommendation for improvement 6: Based on the international review, we propose to explore a hybrid financing (combined fix and performance-based reimbursement) system for the Hungarian HPOs. The system would have two main components: annual or monthly base and project funding. The base funding element covers the monthly operation and horizontal activities of HPOs. In terms of operating costs, it should cover the costs of maintaining the property or rental premises, human resources, equipment necessary to carry out the work and travel costs. The horizontal activities include tasks that are necessary to perform for all HPOs, such as preparing community health plans, networking and cooperation with local actors, and administrative tasks related to the operation of HPOs. Overall, this base funding is a predetermined monthly amount allocated to HPOs to enable the organization to continue to operate and to carry out its core tasks smoothly and continuously.

Recommendation for improvement 7: As a first step, the base funding methodology requires a cost assessment of the HPOs' operational and horizontal activities, which will allow the definition of a baseline cost element for each activity (66). The baseline cost elements for each activity can then be adjusted using at least two factors (such as population and settlement structure) representative of the HPO's area of operation. For example, the amount of base funding may be influenced by the level of human resources, as an HPO with a larger population and more employees in a municipality will receive more resources for wage costs. Alternatively, an HPO with a large number of small, geographically dispersed settlements will receive more funding for travel costs.

Recommendation for improvement 8: The second element, titled project funding is to provide financing and incentives for implementing specific health promotion programmes and services in Hungary. In this context, it is proposed to establish a national health promotion fund to which HPOs can apply for funding. The range of services and programmes eligible for funding should be defined and evaluated by the national professional organization, based on international and local best practices, with mechanisms to review the program portfolio (50). The budget available for each HPO to submit proposals will be allocated based on a multi-component formula. The baseline for the amount an HPO can apply for project funding is the population of the HPO's area of operation, adjusted by the economic development of the area (e.g., per capita spending), the age composition of the population and the health needs of the small district (66). A helpful indicator for determining health needs is the age-adjusted standardized death rate, which combines various health determinants in a single indicator. Its strengths are that it can be calculated at the national and local levels and is comparable internationally with relative ease.

Recommendation for improvement 9: Given the complex and intersectoral nature of public health activities, innovative financing mechanism might be valid to explore. One such tool is outcome-based contracting, used mostly in Anglo-Saxon countries to combine advantages of public and private sector (71–74). This contractual arrangement allows payment after the demonstration the achievement of mutually agreed outcomes. In an outcome-based contracting framework, the focus is on the milestones for payment and the impacts and results achieved by the activities, not the completion of individual activities. This requires investing in monitoring of outcomes as well the development of measurable key performance indicators, which helps to understand the performance of individual HPOs.

##### 3.3.2.3. Legally regulated forms of external funding

In many countries, innovative forms of external financings, such as social impact bonds, individual or company donations, sponsorship, exist to enable organizations to contribute to the financing of public health services and programmes (75–79).

Recommendation for improvement 10: Based on international practices, it would be helpful to have a legally regulated form of external funding available to HPOs and consistent methodological support on how to include external actors and partners in the financing of community services.

To summarize, based on promising international practices, it is recommended to develop the professional management system and networking model further, improve the monitoring and performance assessment capacities, update the service standards and guidelines, and create a flexible operational structure adapted to the population size and characteristics of HPOs. We also recommend using community health plans and health needs assessment, a hybrid financing system, and legally regulated forms of external funding.

## 4. Discussion

The challenges of the twenty-first century require a wellfunctioning public healthcare system, with a focus on prevention, that is complemented by a capable and well -regulated public health system (80). The COVID-19 pandemic has ravaged the Hungarian society, —in some indicators more so than neighboring, and comparably advanced Visegrad countries—indicating the imperative to strengthen the resiliency of the population, and the HPOs can play an integral part in that effort, through community-based health promotion activities (81–83).

The results of our study show that HPOs currently face three main challenges. Perhaps the most crucial challenge is the inconsistent management framework, which can be attributed to several factors. Frequent reorganizations were hindering institutional learning forming of key networks and generation of experienced managers at national level. The lack of welldefined monitoring and performance assessment framework has been one of the weak points of the HPO network since inception (17, 31). In our view, the currently applied key performance indicators, do not generate critical information to assess the impact and outcomes of the services provided by HPOs also lacking to provide valuable insights into the functioning of HPOs. In the absence of an adequate performance assessment system, evidence-based local promising practices cannot be identified, and the impact assessment of domestic adaptation of international best practices by HPOs cannot be carried out. Based on promising international practices, it could be beneficial to improve the professional management system and the monitoring and performance assessment capacities (36, 37, 41, 48, 49). Several studies have also found that it would be beneficial to update and apply the service standards and guidelines and create a flexible operational structure adapted to the population coverage of HPOs (38, 44, 50, 53, 56).

The second, systemic challenge that needs to be addressed is the inflexible financing mechanism, causing several deficiencies. The current financing mechanism does not incentivise for quality service delivery. The current method of base financing is preferable where performance is difficult or almost impossible to measure, yet a specific capacity (such as infrastructure or staff) needs to be provided (70). This method has the advantage of low administration burden and easy planning, but it only encourages quality service delivery if it is linked to a professional quality assurance system, which is lacking currently. Based on promising international practices, developing and operating a hybrid financing system could be beneficial (63, 66, 70). Studies have also found that using community health plans, health needs assessments, and legally regulated forms of external funding would be beneficial (62, 70–75).

The consequence of the first two challenges and their related problems culminates in the third problem—and possibly most severe—the unequal access to HPO services. A significant part of the population still does not have access to these organizations provided free health promotion services. Given the lack of information and evidence to evaluate the results of HPOs so far, it is not easy for policy makers to find arguments for further expansion of the network. To summarize, the deficiencies in management, funding and access to services result in differences in the service portfolio, expertise and activity of HPOs. Heterogeneous training of HPO's staff and incomplete or outdated professional protocols and guidelines result in an inconsistent quality of health promotion programmes. The quality of services and programmes provided is therefore influenced by the motivation and commitment of local HPO staff as well as the commitment of the owning organizations, rather than transparent guidelines.

However, the results of our literature review suggest that it is worth investing resources in improving the management and financing of HPOs. In addition, it may also be worthwhile to increase the financial resources spent on prevention, as the per capita expenditure on prevention in Hungary is considerably below the OECD average (84).

Our study has the following limitations. First, this study identified challenges and problems in the functioning of HPOs based on the literature in Hungarian and an online questionnaire survey based on data and experiences provided by professional and economic managers of HPOs. Potential area for future research, would be to explore the experiences of the population using the services of HPOs, and the experiences of partner organizations of HPOs. Second, there are few methodological and practical guidelines on financing health promotion services available in the international literature, and therefore we have limited information on the practices in individual countries. Finally, this study discusses the financing methodology that should be used to provide the necessary financial resources for HPOs but does not examine the resource generation alternatives for running an HPO system, which could be the subject of future research.

## 5. Conclusion

This study described the current operational and funding system of the Hungarian HPOs, and examined the challenges and problems related to the operation and funding of HPOs. Our results show that the current network of HPOs faces three main challenges: (a) a dysfunctional operating framework, (b) inflexible financing scheme, and lastly but consequently (c) unequal ability to purchase or provide services for the population in their respective jurisdiction. Although HPOs face many challenges, they have become essential in improving local communities' health over the past decade. Further development of the management and funding system for HPOs would bring significant benefits to Hungarian society. For this reason, we formulated our recommendations for improvement based on international experiences.

Based on international experience, it would be appropriate to further develop the management system of HPOs by eliminating the current parallelism at the level of national management and adapting the network operating model and regional management functions. Developing a monitoring system would allow the impact of the services and programmes provided by the HPOs to be monitored, analyzed and evaluated. It may also be appropriate to develop a flexible operating structure for HPOs, where the number of human resources and services provided by the organization is proportional to the number of people living in its area. Based on international experience, it would be worthwhile to establish a unit responsible for developing standards and guidelines for HPO services, updating standards on an ongoing basis and developing knowledge-sharing mechanisms.

Moreover, community health plans and health needs assessments can provide essential input data for financing HPOs, as it is necessary to prepare a cost plan for the programmes and services included in the health plans. The financing of HPOs should be further developed to reach a hybrid financing model. The two main components of this hybrid system would be: an improved base funding would be proposed to cover the operation and horizontal activities of HPOs, considering local attributes such as population epidemiology, socio-economic development. The second component, termed “project financing could be able to provide adequate funding and incentives for the implementation of health promotion programmes and services. In this context, establishing a national health promotion fund is recommended and using outcome-based contracting may be justified. Based on international practice, it would be advisable to make a legally regulated form of external funding available to HPOs (sponsorship, donations).

## Author contributions

KH, GT, CK, BC, and CD: conceptualization and writing—original draft preparation. GT, CD, and KH: methodology. KH, GT, CD, and CK: investigation. BC and CD: resources and data curation. KH and GT: writing—review and editing. GT: visualization. CD: supervision. All authors listed have made a substantial, direct, and intellectual contribution to the work and approved it for publication and have read and agreed to the published version of the manuscript.

## Footnotes

^1^HPO categories were developed based on external criteria, i.e., according to the types of founder and owner organizations defined by law (31).

^2^The funding is fixed at 25 million HUF, we converted it to EUR for ease of understanding, with the exchange rate of 400 HUF/EUR.
