# Supplementary material for: Challenges and opportunities for improvement in the management and financing system of Health Promotion Offices in Hungary
Source: Front Public Health. 2023 Oct 27;11:1219186. doi: 10.3389/fpubh.2023.1219186 (PMC10641462; doi:10.3389/fpubh.2023.1219186)
Supplement: Supplementary file 1 [file Table_1.pdf]

Supplementary Material

Challenges and opportunities for improvement in the management and financing system of Health Promotion Offices in Hungary

Krisztián Horváth, Gergő Túri, Csilla Kaposvári, Borbála Cseh, Csaba László Dózsa

*** Correspondence:**
Krisztián Horváth
horvath.krisztian@med.semmelweis-univ.hu

1. Supplementary Figure S1 Percentage of HPO respondents according to the difficulty of finding staff for specific occupations (easy, difficult, very difficult).

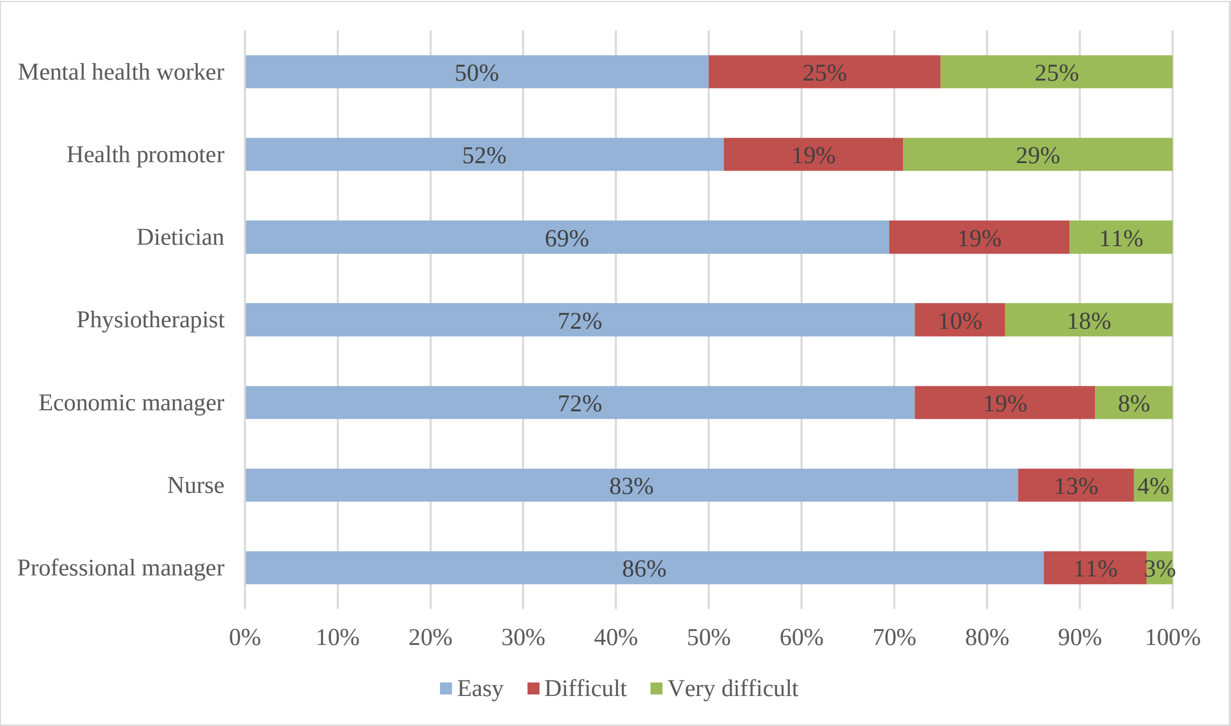

Source: Authors

Supplementary Material

2. Supplementary Figure S2. Percentage of HPO respondents according to the difficulty of organizing specific health promotion programmes (easy, managable, very difficult).

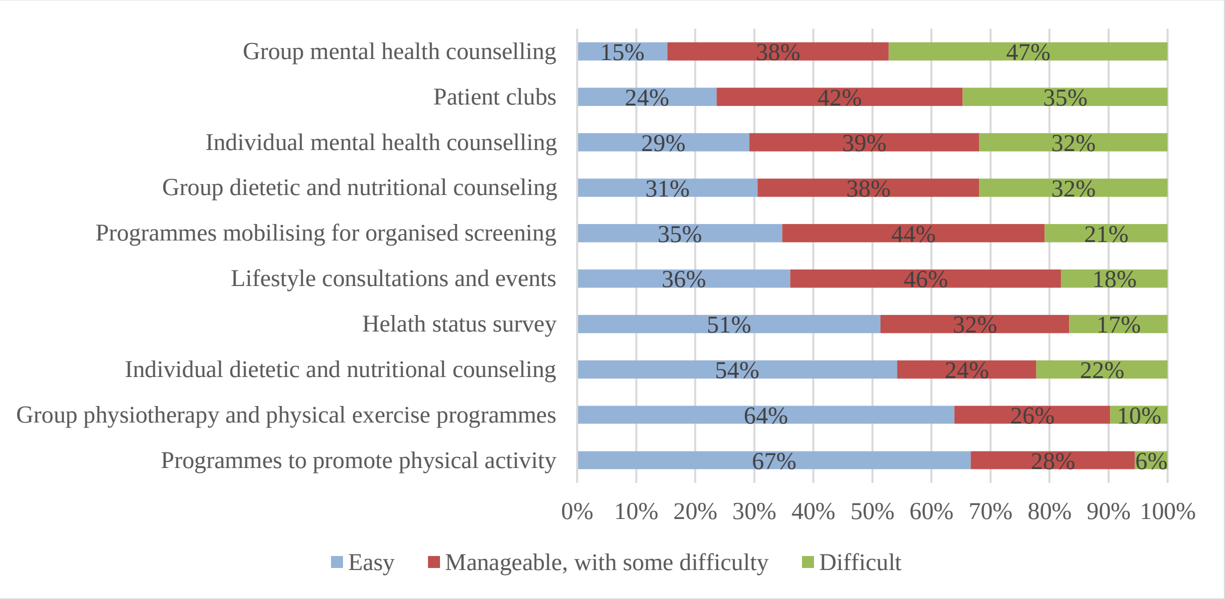

Source: Authors

3. Supplementary Figure S3. Percentage of communication platforms used by HPOs.

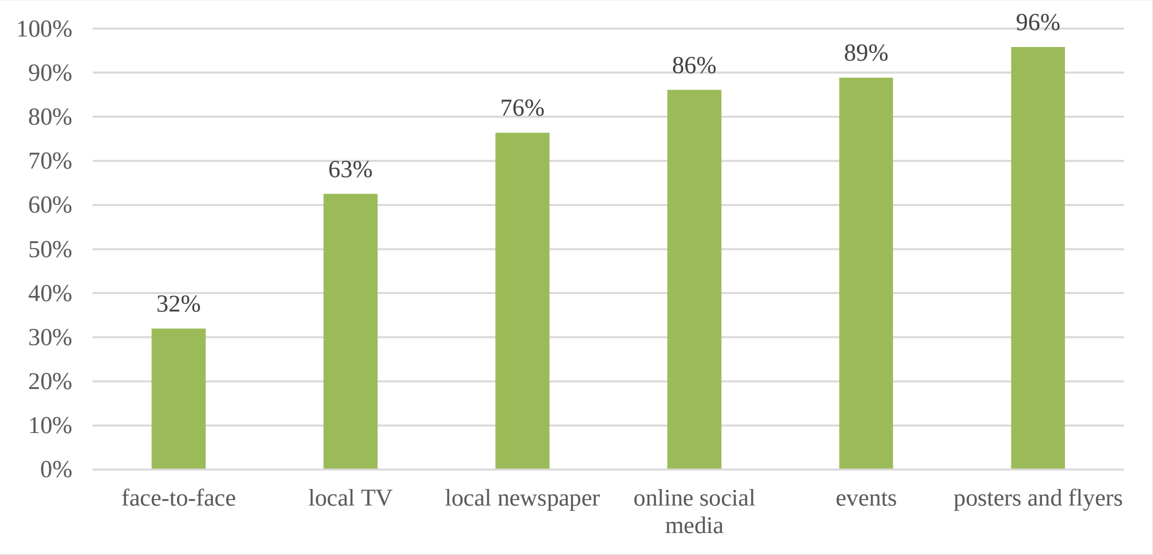

Source: Authors
